# TriCAFFNet: A Tri-Cross-Attention Transformer with a Multi-Feature Fusion Network for Facial Expression Recognition

**DOI:** 10.3390/s24165391

**Published:** 2024-08-21

**Authors:** Yuan Tian, Zhao Wang, Di Chen, Huang Yao

**Affiliations:** Faculty of Artificial Intelligence in Education, Central China Normal University, Wuhan 430079, China; tianyuan_ccnu@163.com (Y.T.); wangzhao@mails.ccnu.edu.cn (Z.W.); chendi@ccnu.edu.cn (D.C.)

**Keywords:** facial expression recognition, vision transformer, multi-feature, tri-cross attention

## Abstract

In recent years, significant progress has been made in facial expression recognition methods. However, tasks related to facial expression recognition in real environments still require further research. This paper proposes a tri-cross-attention transformer with a multi-feature fusion network (TriCAFFNet) to improve facial expression recognition performance under challenging conditions. By combining LBP (Local Binary Pattern) features, HOG (Histogram of Oriented Gradients) features, landmark features, and CNN (convolutional neural network) features from facial images, the model is provided with a rich input to improve its ability to discern subtle differences between images. Additionally, tri-cross-attention blocks are designed to facilitate information exchange between different features, enabling mutual guidance among different features to capture salient attention. Extensive experiments on several widely used datasets show that our TriCAFFNet achieves the SOTA performance on RAF-DB with 92.17%, AffectNet (7 cls) with 67.40%, and AffectNet (8 cls) with 63.49%, respectively.

## 1. Introduction

Facial expression is one of the most prevalent and vital signals conveying human emotions and intentions. As one of the fundamental tasks in computer vision, facial expression recognition (FER) has attracted increasing attention in recent years due to its close relevance to human–computer interaction, education, healthcare, and online monitoring applications.

Two types of datasets are primarily used in the facial expression recognition task. The first type is datasets in laboratory environments such as JAFFE [1], CK+ [2], etc. The facial expression images in these datasets are pictures of individuals making expressions in a laboratory environment according to instructions and then being captured by a camera. The second type is datasets in real-world scenarios, such as RAF-DB [3], AffectNet [4], FERPlus [5], etc. Most of the images in these datasets are collected from the Internet and obtained by keyword searches. The background environments in each picture vary significantly. In the early studies on facial expression recognition, datasets primarily used were collected in laboratory settings. For these datasets, researchers relied on handcrafted features such as HOG (Histograms of Oriented Gradients) [6], LBP (Local Binary Patterns) [7], LDP (Local Directional Patterns) [8], and SIFT (Local Scale-Invariant Feature Transform) [9] features to analyze facial expressions, and they achieved good recognition results. However, in practical applications, the environment is complex and dynamic. Using handcrafted features for facial expression recognition faces challenges due to uncontrollable factors such as occlusion, head posture variations, facial deformation, etc., leading to a decrease in recognition effectiveness. With the development of deep learning, CNNs (convolutional neural networks) have been introduced into the field of facial expression recognition due to their efficient computational efficiency and powerful feature extraction capabilities [10,11,12,13]. Algorithms based on CNNs have achieved outstanding results on datasets in laboratory environments. However, constrained by the limitations of the local receptive fields, the CNNs fail to fully consider the importance of the global information of images. Although the recognition accuracy on datasets in a laboratory environment has already exceeded 99%, the recognition accuracy on datasets in a natural environment still needs to be improved. After the Transformer was applied to computer vision, Xue et al. [14] designed the first Transformer network, TransFER, for facial expression recognition, which improved Vision Transformer (ViT) by combining global and local information. Since then, ViT has been introduced to facial expression recognition tasks with state-of-the-art (SOTA) results [15,16,17,18,19,20].

Meanwhile, researchers have explored the role of different features in facial expression recognition. LBP features can effectively describe the texture information of an image and are robust to illumination changes, which can be a complement of image features. By combining LBP features with image features and fusing the global and local information, facial expression recognition performance can be effectively improved [19,21,22]. Some studies have investigated the role of facial landmarks in facial expression recognition. Landmarks are a set of crucial points in a face image, which can exclude the interference of factors such as skin color, gender, age, and image background and have a specific generalization ability. They provide a sparse representation of the facial region and can be used as a complement to image information. Landmark features extracted automatically from landmarks can help the model to focus on more important details, which can help to improve the variance value of the inter-class differences [23,24]. Zheng et al. [25] proposed the idea of fusing landmark features and image features. They found that landmark features are the key to solving the inter-class similarity and intra-class differences. The image features are used as supplementary information to assist landmark features in facial expression classification. Although this approach has achieved good results, it still has the following problems. On the one hand, adding scale information solely through upsampling and downsampling may not enrich semantic details as much as introducing other features. On the other hand, the network structure introduces a large number of parameters, leading to increased computational cost.

Despite the outstanding performance of these algorithms, the following problems still exist: (1) Insufficient feature information. Convolutional neural networks can only extract the CNN feature of images, and the input of ViT can only be a single RGB image. Both can only handle single pieces of information, but they fail to make effective use of features that help distinguish subtle differences between images. (2) Inter-class similarity. There is a similarity between pictures of the same person in different expressions, and this similarity makes it difficult to distinguish between different expressions. There are similarities between different expression categories for the same person, posing a challenge in distinguishing between different expressions. (3) Intra-class variability. There may be significant variability between images of the same expression from different individuals, and this variability can easily lead to the misrecognition of expression categories. For example, differences in skin color, gender, image environment, and age among different individuals can contribute to such variations. (4) Model parameters and floating-point operations per second (FLOPs). Many works have only considered accuracy as the model evaluation metric. However, a large number of parameters and slow operation speed cannot meet the needs of practical applications, especially the requirements of real-time expression recognition tasks. Therefore, it is essential to include model parameter size and FLOPs in the evaluation criteria.

In this paper, we propose a tri-cross-attention transformer with a multi-feature fusion network (TriCAFFNet) to solve further the four problems of insufficient feature information, inter-class similarity, intra-class variability, and the excessive number of model parameters. TriCAFFNet is a model that processes landmark features, CNN features, and LBPHOG (fusion features of Local Binary Pattern and Histogram of Oriented Gradients) features by using the original input image and the fused feature image as model inputs. Based on the excellent performance of the facial expression recognition of LBP and HOG features, they can be used as important information to distinguish different faces, thus decreasing the intra-class variability instead of other handcraft features. The proposed model uses the a convolutional neural network to extract high-level features from the fused image of LBPHOG, enhancing the model’s ability to recognize facial expressions and improving overall robustness. With the advantage of landmark features in automation and robustness in the unexpected surrounding factors of an image, landmark features are used to distinguish the subtle differences and decrease the inter-class similarity. Meanwhile, the CNN features are used as complementary information for the landmark features and LBPHOG features, and the proposed tri-cross-attention mechanism can adaptively fuse these three types of feature information. In addition, our model extracts two different features using the same image backbone, which keeps the total number of parameters of the model at a low level while ensuring recognition accuracy at an advanced level. In summary, the contributions of this paper are as follows:

1. This paper proposes to introduce LBP, HOG, landmark, and CNN features of an image simultaneously in the facial expression recognition task to alleviate the problem of insufficient feature information in the expression recognition task by fusing and cross-utilizing multiple different features.
2. A tri-cross-attention mechanism is proposed, which enhances the model’s ability to resolve inter-class similarities and intra-class variability by guiding the three types of features to each other, adaptively fusing the three types of features, and comprehensively exploiting the advantages of each feature.
3. The effectiveness of TriCAFFNet was verified through extensive experiments, with TriCAFFNet achieving state-of-the-art recognition accuracies on two commonly used datasets (RAF-DB 92.17%, AffectNet (7 cls) 67.40%, AffectNet (8 cls) 63.49%).

## 2. Related Work

### 2.1. Facial Expression Recognition in the Wild

The task of facial expression recognition in real-world scenarios faces unknown environmental conditions. Traditional methods based on texture feature extraction (e.g., HOG, LBP, LDP, SIFT, etc.) are susceptible to the interference of real-world environmental factors, which affects their robustness and accuracy in practical applications. These methods mainly rely on the texture information of the image to capture facial expression features. Still, the common factors in the natural environment, such as lighting changes, posture changes, and occlusions, can interfere with the texture of the image, thus affecting recognition.

To address these issues, researchers are increasingly turning to deep learning methods to enhance the performance of facial expression recognition. The most successful deep learning methods among them are based on CNNs. They are able to automatically learn high-level abstract features with solid robustness to factors such as illumination changes, pose changes, and local occlusion. By training on large-scale datasets, essential features of facial expressions can be effectively extracted. Standard deep learning-based methods for facial expression recognition utilize CNNs for feature extraction and combine them with other classifiers, such as support vector machines, multilayer perceptrons, etc., for expression classification. For example, Savchenko et al. [26] investigated lightweight convolutional neural networks for facial expression recognition and verified the effectiveness of CNNs for the task of facial expression recognition. Tang [27] replaced the softmax layer of a CNN with support vector machines (SVMs) for facial expression recognition, achieving a significant improvement in performance. They chose to use lighter-weight network models or replace components with more minor parameter counts to keep the overall model’s parameter count at a lower level. Vo et al. [28] proposed a super-resolution pyramid network structure that addresses issues related to pose variations, head orientation changes, and image resolution in practical applications. With the significant success of ViT in the field of computer vision, many researchers began to pay attention to the application of ViT in facial expression recognition tasks. Xue et al. [29] proposed the TransFER model and explored the significance of Transformers in facial expression recognition. The model effectively describes the relationships between different facial regions by introducing ViT-FER and a multi-head self-attention dropout algorithm. VTFF [19] utilizes attention selective fusion (ASF) to merge two feature maps generated by two branches of CNN, capturing discriminative information with global-local attention on multiple features. Meanwhile, VTFF utilizes the global self-attention mechanism to model the relationship between image patches so that the network model can adapt to the facial expression recognition task in the natural environment. Li, Huihui. et al. [30] introduced LCFC using two modules inspired by human cognitive traits: the Adaptive Relative Transformation (AFRT) and Adaptive Graph Convolutional Network (AFGCN). The AFRT module creates new cognitive features based on relative positions, while the AFGCN module leverages interactions among expression categories. Together, these modules improve FER classification performance significantly, achieving state-of-the-art results across various datasets.

### 2.2. Feature Fusion for FER

In facial expression recognition, the input information of the model is equally important compared to the model’s structure. More high-level and discriminative features can help the model better distinguish the subtle differences between facial expressions. Feature fusion aims to integrate different types of feature information, effectively enhancing the generalization ability and recognition performance of the model.

In recent years, many research works have focused on enhancing the accuracy and robustness of facial expression recognition using feature fusion methods. A commonly used feature fusion method is multimodal fusion, which combines features from different sensors or modalities. For example, Chen et al. [31] proposed a method that combines dynamic texture, geometric features, and acoustic features to address facial expression recognition in natural environments. By integrating facial morphological features with acoustic emotional features, they obtained a more comprehensive representation of facial expressions. A commonly used feature fusion method is combining the color features, texture features, and depth features of facial images to capture facial expression details at different levels. Shao and Qian [32] proposed a two-branch CNN to extract LBP features and depth features in parallel, fusing the two feature maps through a concatenation method. Another commonly used feature fusion method is cascade fusion, which improves recognition performance by cascading multiple classifiers or feature expressions. This method can capture complex facial dynamics by hierarchically transmitting and integrating feature representations, for example, by cascading CNNs to extract low-level features and then using Long Short-Term Memory (LSTM) or attention mechanisms to learn higher-level dynamic features [22,23]. In addition, some research works have focused on channel-level feature fusion. By concatenating or performing weighted merging on the feature maps at the channel level from different convolutional layers, the network can provide a more comprehensive and diverse feature representation. Yang et al. [18] introduced an attention mechanism to adaptively select, strengthen, and suppress the feature channels. Experimental results demonstrate that this method is also a practical feature fusion approach. In addition to the above methods, several studies have explored the use of techniques such as meta-learning [33], generative adversarial networks (GAN) [34], and transfer learning [35] for feature fusion. These methods learn complex mapping relationships or migrate prior knowledge to obtain feature representations that are more discriminative and generalizable. Through feature fusion, the model acquires rich feature information. Building upon this foundation, we believe that targeted features are crucial for better problem-solving. For instance, LBP and HOG features, due to their rich texture information, effectively distinguish between different faces. Introducing them enhances the model’s ability to differentiate between different faces and alleviates inter-class similarity issues. Additionally, landmark features significantly distinguish subtle local changes in faces, helping the model mitigate intra-class variability. In this paper, by fusing landmark features, LBPHOG fusion features, and CNN features, more abundant information is provided to the model.

### 2.3. Vision Transformer

In recent years, the Transformer architecture has garnered significant interest across diverse deep-learning applications, particularly in natural language processing. Introduced by [36], it was initially tailored for sequence-to-sequence tasks. This architecture comprises stacked encoder and/or decoder layers, enabling efficient and scalable processing of extensive input data, thereby facilitating the learning of intricate patterns within the data. Vision transformer (ViT) has been applied in the field of computer vision with great success with its excellent ability to capture long-range dependencies on large-scale datasets. Dosovitskiy et al. [37] first introduced Transformers from natural language processing to computer vision. ViT segments the image into many small pieces and then computes the relationship between different regions through a self-attention mechanism. This approach achieved impressive performance in image classification, target detection, and semantic segmentation.

ViT is also widely used in facial expression recognition. Li, H. et al. [17] proposed a new Transformer-based method, mask vision transformer (MVT), which is used for facial expression recognition tasks in natural environments. It consists of a Transformer-based mask generation network (MGN) for generating facial image masks capable of filtering complex backgrounds and occlusions and a dynamic relabeling module for correcting false labels in the FER dataset. Li, Y. et al. [38] introduced the SPWFA-SE model for facial expression recognition, combining slide patches for comprehensive local feature extraction without landmarks, attention modules to highlight critical regions, and SE blocks for optimizing feature representation. Extensive testing across diverse datasets demonstrated state-of-the-art performance and superior generalization, with the model also showing efficiency benefits through reduced parameters and faster training times compared to existing approaches. Zheng et al. [25] designed a cross-fusion method based on Transformer. The model can efficiently leverage facial landmark features and image features to maximize the focus on significant facial regions. This approach simultaneously addresses inter-class similarity, intra-class variation, and scale sensitivity within a unified framework. Our TriCAFFNet model introduces multi-feature fusion and the tri-cross-attention mechanism. By synthesizing the strengths of different features, it effectively addresses the challenges in facial expression recognition, demonstrating excellent performance in experiments.

## 3. Method

### 3.1. Baseline

**Figure 1:**
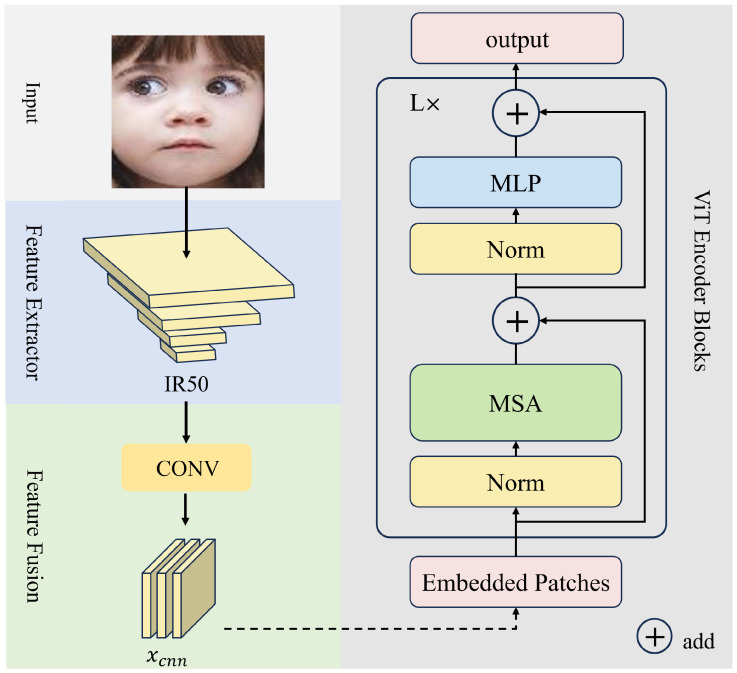
The architecture of Baseline.

Figure 1 illustrates the overall architecture of our Baseline model. For the input image, we obtain the feature matrices $X_{cnn}\in R^{P\times D}$ with an image backbone IR50 [39], where *P* is the number of patches and *D* is the feature dimension. After obtaining the image features $X_{cnn}$, the transformer architecture utilizes a self-attention mechanism to capture correlations across patches, achieved by the Multi-head Self-Attention Layer (MSA) in the transformer architecture. The input $X_{cnn}$ is first mapped to three matrices: the query matrix *Q*, key matrix *K*, and value matrix *V* by linear transformations:

(1) $$Q=X_{cnn}W_{Q},K=X_{cnn}W_{K},V=X_{cnn}W_{V}$$

where $W_{Q},W_{K},W_{V}\in R^{D\times D}$.

The vanilla transformer attention block can be described by the following equation:

(2) $$Attention\left(Q,K,V\right)=Softmax\left(QK^{T}/\sqrt{d}\right)V$$

where $\sqrt{d}$ is the scaling factor for appropriate normalization.

Then, the encoder output is calculated by the vanilla transformer encoder, which consists of MSA and MLP with a layer normalization operator. The calculation can be described as the following two equations. Finally, the encoder is sent into the output layer with an SE block and another MLP to obtain the likelihood result.

(3) $$X_{cnn}^{\prime }=MSA\left(Q,K,V\right)+X_{cnn}$$

(4) $$X_{cnn\_out}=MLP\left(Norm\left(X_{cnn}^{\prime }\right)\right)+X_{cnn}^{\prime }$$

### 3.2. TriCAFFNet

**Figure 2:**
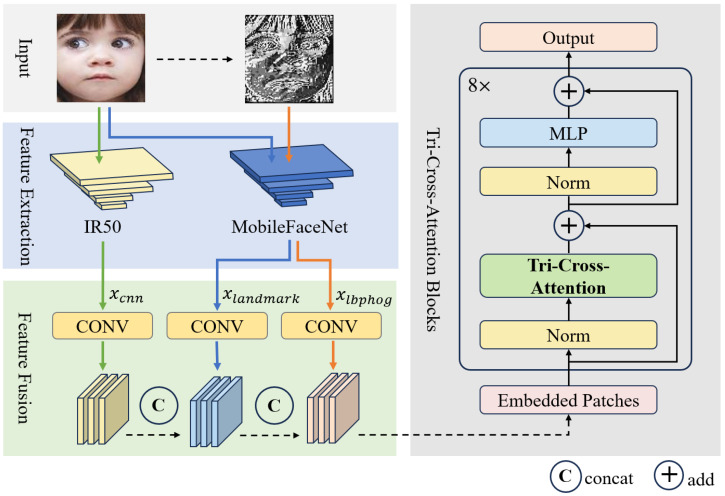
The overall architecture of TriCAFFNet, a facial landmark detector MobileFaceNet, is applied to obtain landmark features and the advanced LBPHOG features, and an image backbone IR50 is used to extract image features.

Figure 2 illustrates the overall architecture of TriCAFFNet. Our network structure includes a multi-feature input, a feature extraction block, a feature fusion block, several tri-cross-attention blocks, and an output. The multi-feature input comprises two types of images: the original facial image and the fused LBPHOG feature image. The fused LBPHOG feature image is generated by processing the original facial image using the LBP and HOG methods. In the feature extraction block, the original face image and the fused LBPHOG feature image are first preprocessed and then inputted into the feature extraction block to obtain the LBPHOG feature map, the CNN feature map, and the landmark feature map. The network contains eight tri-cross-attention blocks. For computing cross-attention maps, the Q-matrices of different features are swapped, and the Q-matrices of the other two features are merged to represent the Q-matrix of the CNN feature in each block. The cross-attention maps are spliced to obtain the fused cross-attention map, which is processed by the SE-block and fully connected layers to obtain the final expression probability output. In the following section, we discuss the characteristics of different features and how they exchange information.

### 3.3. Multi-Feature

Feature fusion is the key to enhancing the ability to solve inter-class similarities and intra-class variability in facial expression recognition. Obtaining high-level semantic information for expression recognition through feature fusion is a critical way to improve the accuracy of expression recognition. LBP features and HOG features have been widely used in face recognition and have achieved good results. LBP features can express the texture information of an image, and HOG can express the edge information of an image. They have similar characteristics to landmarks, which can provide significant information on critical parts of a face to support the distinction of subtle differences between similar faces. HOG features can enhance the edge information of the LBP features, and by fusing the two feature images, the texture features and edge features can be fused within a single image. In this paper, the improved circular LBP feature extraction algorithm and HOG feature extraction algorithm are used to extract the LBP feature image and HOG feature image of the input RGB image, respectively. The two feature images are fused by element-wise addition, followed by further extraction of their advanced features to improve the model’s capability to distinguish the critical areas of the face.

Meanwhile, the global information of the image is also essential for facial expression. We extract the CNN features of the input RGB image using a convolutional neural network and combine the local and global information of the picture. In the process of extracting the above features, the MobileFaceNet network pre-trained by ImageNet [40] is used as the landmark detector, and the parameters are frozen during the training process without parameter updating in order to extract the landmark features quickly. In addition, taking advantage of MoblieFaceNet’s sensitivity to salient regions, we use the same MobileFaceNet network as a feature extractor to extract high-level features from LBPHOG feature images. The experimental results also demonstrate that this not only reduces the overall number of parameters of the model but also extracts useful feature information. The IR50 network pre-trained by Ms-Celeb-1M [41] dataset is used as the Image Backbone to extract CNN features of the input RGB image. Three feature matrices $X_{landmark}\in R^{P\times D}$, $X_{lbphog}\in R^{P\times D}$, and $X_{cnn}\in R^{P\times D}$ are extracted, containing landmark feature, LBPHOG high-level feature, and CNN feature.

### 3.4. Tri-Cross-Attention

For the input to the Transformer encoder $X_{landmark}\in R^{P\times D}$, $X_{lbphog}\in R^{P\times D}$, and $X_{cnn}\in R^{P\times D}$, the three streams of inputs are converted into the query matrix *Q*, the key matrix *K*, and the Value matrix *V*, respectively, by linear transformation:

(5) $$Q_{1}=X_{landmak}W_{Q1},K_{1}=X_{landmark}W_{K1},V_{1}=X_{landmark}W_{V1}$$

(6) $$Q_{2}=X_{cnn}W_{Q2},K_{2}=X_{cnn}W_{K2},V_{2}=X_{vnn}W_{V2}$$

(7) $$Q_{3}=X_{lbphog}W_{Q3},K_{3}=X_{lbphog}W_{K3},V_{3}=X_{lbphog}W_{V3}$$

where $W_{Q1}$, $W_{Q2}$, $W_{Q3}$, $W_{K1}$, $W_{K2}$, $W_{K3}$, $W_{V1}$, $W_{V2}$, $W_{V3}$$\in R^{D\times D}$.

**Figure 3:**
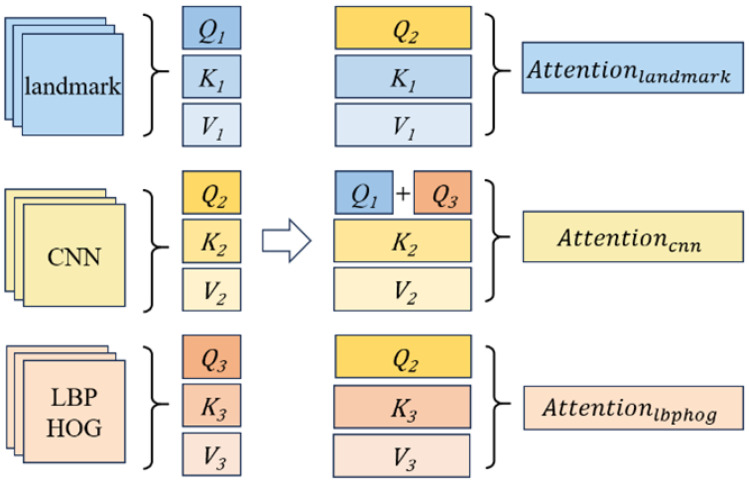
The architecture of tri-cross-attention module.

The architecture of the tri-cross-attention module is shown in Figure 3, which can be described as the following formulas:

(8) $$Attention_{landmark}=Softmax\left(Q_{2}K_{1}^{T}/\sqrt{d}\right)V_{1}$$

(9) $$Attention_{cnn}=Softmax\left(\left(Q_{1}+Q_{3}\right)K_{2}^{T}/\sqrt{d}\right)V_{2}$$

(10) $$Attention_{landmark}=Softmax\left(Q_{2}K_{3}^{T}/\sqrt{d}\right)V_{3}$$

where $\sqrt{d}$ is the scaling factor used for normalization. $Q_{1}$, $Q_{2}$, and $Q_{3}$ are matrices computed from the landmark feature map, CNN feature map, and LBPHOG feature map, respectively.

During the computation of the respective attention, the *Q* matrix of the CNN feature map is replaced with $Q_{1}+Q_{3}$, and the *Q* matrix of the other two feature maps is replaced with $Q_{2}$. The high distinguishing ability of landmark feature maps and LBPHOG high-level feature maps is used to guide CNN features in calculating self-attention. At the same time, the information on landmark features, LBPHOG high-level features, and CNN features is preserved. The tri-cross-attention module introduces global information by exchanging the *Q* matrix of the CNN features into the attention computation process of the landmark features and the LBPHOG high-level features, respectively. For the given inputs $X_{landmark}$, $X_{lbphog}$ and $X_{cnn}$, their computation through the encoder can be expressed as the following equation:

(11) $$X_{landmark}^{\prime }=CMSA_{landmark}\left(Q_{2},K_{1},V_{1}\right)+X_{landmark}$$

(12) $$X_{landmark\_out}=MLP\left(Norm\left(X_{landmark}^{\prime }\right)\right)+X_{landmark}^{\prime }$$

(13) $$X_{cnn}^{\prime }=CMSA_{cnn}\left(Q_{1},Q_{2},Q_{3},K_{2},V_{2}\right)+X_{cnn}$$

(14) $$X_{cnn\_out}=MLP\left(Norm\left(X_{cnn}^{\prime }\right)\right)+X_{cnn}^{\prime }$$

(15) $$X_{lbphog}^{\prime }=CMSA_{lbphog}\left(Q_{2},K_{3},V_{3}\right)+X_{lbphog}$$

(16) $$X_{lbphog\_out}=MLP\left(Norm\left(X_{lbphog}^{\prime }\right)\right)+X_{lbphog}^{\prime }$$

where CMSA(·) denotes cross-attention multi-head self-attention block, Norm(·) denotes normalization operation and MLP(·) denotes multi-layer perceptron.

In the process of fusing two *Q* matrices in the CNN stream, we explored different fusion methods, including direct element-wise addition, concatenation followed by dimension reduction using a 1 × 1 convolution, and upsampling or downsampling approaches. Experimental results show that element-wise addition is the most effective. We believe that in the process of summing the two matrices, the fusion matrix is able to obtain the components of the two sub-matrices with large weight values, fusing the more important attention components of each of the two sub-matrices.

## 4. Experiments

### 4.1. Datasets

The availability of large-scale datasets in real-world scenarios is essential for facial expression recognition tasks. Large-scale datasets can provide rich samples of facial expressions, which can help networks learn more comprehensive features. These datasets can cover face images of different ages, genders, races, and emotional states, thus making the trained models more robust in real-world scenarios. In this paper, two commonly used facial expression datasets in real-world scenarios are chosen to validate the effectiveness of the proposed TriCAFFNet model.

RAF-DB [3]: Real-world Affective Faces Database (RAF-DB) is a facial expression recognition dataset containing seven different basic facial expressions (happy, sad, anger, surprise, fear, disgust, and neutral). The dataset includes 29,672 face images from the real world. Among them, 15,339 images are used for facial expression recognition tasks, with 12,271 images for training and 3068 images for testing. Each image is annotated with labelled expression categories and intensity levels. The RAF-DB dataset is widely used in the field of facial expression recognition and is one of the commonly used benchmark datasets for evaluating and comparing different algorithms.

AffectNet [4]: AffectNet is one of the largest publicly available datasets for facial expression recognition tasks. It is a large-scale dataset in a real-world scenario, containing more than one million facial images collected from the Internet. The photos are labelled into eight emotion categories, including the seven primary expressions and the contempt expression. Comparison of model recognition results can be based on two different uses: accuracy based on seven emotion categories and accuracy based on eight emotion categories. We verify the recognition effect of the proposed model based on these two uses.

### 4.2. Implement Details

In the experiments, a standardized preprocessing is applied to all input images, including resizing to a uniform size of 224 × 224, random horizontal flipping, random vertical flipping, random addition of Gaussian noise, and random erasure of specific regions in the images. The feature extraction model utilizes the IR50 model pre-trained on the Ms-Celeb-1M dataset as the image backbone. The MobileFaceNet, with the same frozen training parameters, is employed to extract facial landmarks and high-level LBPHOG features. The learning rate is initialized to 0.0004. Adam optimizer is utilized. The batch size is set to 200. The mlp ratio and drop path rate are set to 2.0 and 0.01, respectively. The cross-entropy loss function is used as the loss function. We implemented all the experiments on an NVIDIA RTX 3090 GPU based on the Pytorch framework.

### 4.3. Comparison with State-of-the-Art Results

In this section, we compare the recognition results of the method proposed in this paper with some SOTA methods on RAF-DB and AffectNet datasets.

Results on RAF-DB: We compare the results with the FER algorithms that have achieved SOTA performance on the RAF-DB dataset in recent years. The results are shown in Table 1. The experimental results indicate that our TriCAFFNet achieved SOTA performance on the RAF-DB dataset with an accuracy of 92.17%. It outperforms MVT (88.62%) [17], PSR (88.98%) [28], QOT (89.97%) [42], TransFER (90.91%) [14], APViT (91.98%) [29], and LCFC(89.23%) [30] by 3.55%, 3.19%, 2.2%, 1.26%, 0.19%, and 2.94%, respectively. It surpasses our Baseline(91.88%) by 0.29%, and also surpasses the second-highest method, POSTER (92.05%) [25], by 0.12%.

Results on AffectNet (7 cls): The results of TriCAFFNet on AffectNet (7 cls) dataset in comparison with the previous methods are presented in Table 2. TriCAFFNet (67.40%) outperforms MVT (64.57%) [17], EAC (65.32%) [43], TransFER (66.23%) [14], APViT (66.91%) [29], and POSTER (67.31%) [25] on AffectNet (7 cls) by 2.83%, 2.08%, 1.17%, 0.43%, and 0.09%, respectively. It surpasses our Baseline(66.34%) by 1.06%, and also surpasses the second-highest method, QOT (67.37%) [42], by 0.03%.

Results on AffectNet (8 cls). Table 3 shows the results of TriCAFFNet on the AffectNet (8 cls) dataset in comparison with the previous methods. TriCAFFNet (63.49%) is 2.81%, 2.16%, 2.09% 1.32% higher than PSR (60.68%) [28], ARM (61.33%) [44], MVT (61.40%) [17] and LCFC (62.17%) [30], respectively. It surpasses our Baseline(63.14%) by 0.35% and is 0.15% higher than the second highest POSTER (63.34%) [25] method.

**Figure 4:**
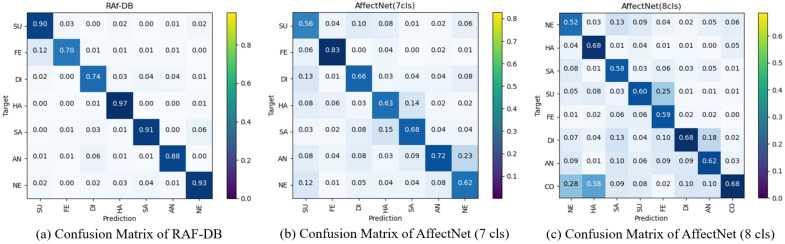
Confusion matrics of TriCAFFNet on RAF-DB, AffectNet (7-cls) and AffectNet (8-cls).

As shown in Figure 4, we display the confusion matrices of the proposed model on these datasets. The darker colors of the diagonal positions in the confusion matrix represent the higher recognition accuracy of the class. Lighter colors in other positions indicate lower misidentification rates. It can be seen that TriCAFFNet performs well on the RAF-DB dataset and is weaker than the different classes, only in Fear and Disgust, which have fewer training samples. It also reaches a good level on AffectNet.

Model size and FLOPs: In model evaluation, the number of parameters and FLOPs are also crucial metrics. Table 4 compares the number of parameters and FLOPs of our TriCAFFNet model with MVT [17], VTFF [19], Transfer [14], POSTER [25] and APViT [29]. The proposed model keeps the number of parameters at a lower level by using the same image backbone to extract different features and also improves performance.

### 4.4. Result Analysis

In order to better understand TriCAFFNet’s efficient use of multiple features and the effectiveness of the architecture, we compared TriCAFFNet with other SOTA methods in terms of facial expression recognition accuracy as well as average accuracy on the seven classes of expressions in the RAF-DB dataset. The results are shown in Table 5.

Among the seven expression categories, TriCAFFNet achieves the highest facial expression recognition accuracy in neutral and sad compared to all other models listed in the table. Neutral expressions are easily misidentified as other expressions because they have no apparent emotional coloring. TriCAFFNet outperformed the second-ranked model TransFER [14] on neutral by 2.79%, which indicates that TriCAFFNet is capable of utilizing multiple features to distinguish subtle differences between various expressions, enhancing its ability to address inter-class similarity issues. The feature differences between sad and other expressions are pretty obvious. TriCAFFNet enhances this distinctiveness by introducing landmark features and LBPHOG features, thereby improving the model’s ability to address inter-class differences. Additionally, all models perform the worst in the fear category, which is because, in the training set of RAF-DB, the number of images in the fear category accounts for only 2.2% of the total number of images. It is nearly 20 times lower than the happy (38.9%) category, which has the most significant proportion. Therefore, the model lacks a sufficient number of training samples for the fear category. TriCAFFNet performs well in this category as well, which indicates that even with a limited number of training samples, TriCAFFNet is able to capture various features that provide valuable information for recognition.

We further analyze the misrecognition rates of the model on the neutral, fear, and sad expression categories and compare them with POSTER [25]. The results, as shown in Table 6, illustrate the ability of TriCAFFNet to address both inter-class similarity and intra-class variability issues. In recognition of the neutral category, the probability of TriCAFFNet misidentifying it as fear or anger is 0, and the likelihood of misidentifying it as other expression categories is also maintained at a low level. Compared to POSTER [25], TriCAFFNet can further reduce the probability of misidentifying neutral as happy, surprise, and disgust. In the category of fear, which has the smallest sample size, the number of misidentifications as surprise and sad is relatively high due to the dataset itself. However, TriCAFFNet is still able to keep the number of misidentifications of fear as other categories, excluding surprise and sad, at a low level.

**Figure 5:**
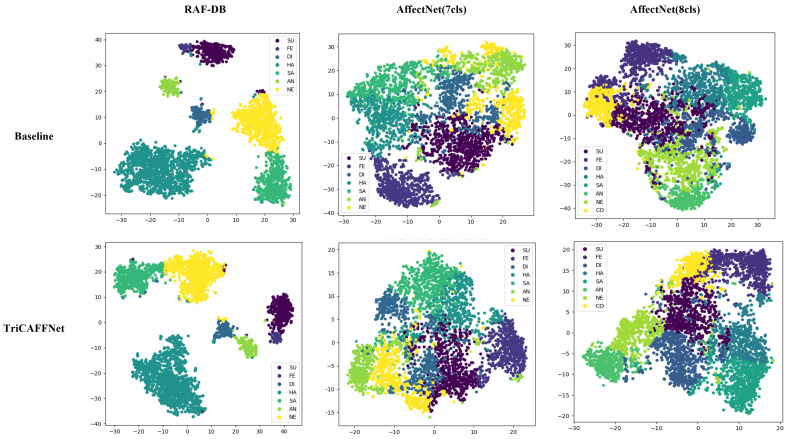
Visualization of high dimensional space t-SNE visualization results on RAF-DB and AffectNet.

As shown in Figure 5, the high-dimensional features of TriCAFFNet are visualized using the t-SNE [45] method. t-SNE visualization plots of the RAF-DB dataset present apparent clustering effects and obvious separability, where the points of different colors are far away from each other. In contrast with our Baseline model, the points of the same color are tightly clustered in the low-dimensional space, which suggests that they have similar features in the high-dimensional space, thus further illustrating the SOTA capability of TriCAFFNet in performing the expression classification task. The AffectNet dataset, because of its massive number of samples and the unbalanced distribution among the samples, has closer distances between the points of individual colors. TriCAFFNet also presents a competitive performance.

### 4.5. Ablation Study

We conducted ablation experiments on RAF-DB and AffectNet datasets to validate the effectiveness of our proposed architecture, and the results are shown in Table 7.

Landmark. We verify the importance of landmark features by removing landmark features in the three-stream attention mechanism. After removing landmark features, the recognition accuracy of TriCAFFNet on the RAF-DB dataset and AffectNet dataset decreases from 92.17% and 67.40% to 91.40% and 67.10%, and its recognition effect is greatly reduced. Therefore, we introduce landmark features to enhance the ability of the model to distinguish the subtle differences between images, which can effectively enhance the recognition ability of the model.

LBPHOG. We investigated the effect of the introduction of LBPHOG features on the recognition effect. It can be found that the recognition rate of the model on both datasets decreases when the LBPHOG features are removed (RAF-DB decreases by 0.43% and AffectNet decreases by 0.10%). Therefore, the ability of the model to distinguish subtle differences between images can be further enhanced by introducing LBPHOG features, thus improving the accuracy of facial expression recognition.

Tri-Cross-Attention. We explore the effect of the tri-cross-attention mechanism on the model recognition results by removing it. After removing the tri-cross-attention module, the accuracy decreases by 0.78% on RAF-DB and 0.26% on AffectNet. The recognition performance was the poorest in the three ablation experiments, which indicates that the proposed tri-cross-attention mechanism is crucial for the model. For three different features, the tri-cross-attention mechanism enables mutual fusion and guidance. Compared with the common fusion method, it is more helpful in improving the recognition accuracy of the model.

## 5. Conclusions

In this paper, we propose the tri-cross-attention Transformer with a multi-feature fusion network (TriCAFFNet) for facial expression recognition. We propose to improve the model’s ability to solve the inter-class similarity and the intra-class variability problems by introducing landmark features, LBPHOG features, and CNN features of the image to enable the model to acquire sufficient information related to recognition. The proposed tri-cross-attention mechanism is used to make the three features fuse and guide each other. A large number of FER experiments show that TriCAFFNet achieves SOTA performance while keeping the number of parameters as well as FLOPs at a low level, which makes TriCAFFNet a better choice for FER as it strikes a good balance between accuracy and computational complexity.

We enhanced the model’s recognition capability by selectively leveraging distinct features. However, this approach also increased the complexity of our model inputs, necessitating additional preprocessing steps. Furthermore, during feature extraction, we employed a single parameter-frozen backbone to extract advanced features from two different types of features, significantly reducing the number of trainable parameters and simplifying the model’s complexity. Nonetheless, the inclusion of two backbones and a tri-cross-attention ViT means there is still room for improvement in optimizing the overall parameter count despite maintaining it at a relatively low level.

## Figures and Tables

**Table 1:** Comparison results with other SOTA FER algorithms on RAF-DB.

Methods	Year	Accuracy
PSR	CVPR 2020	88.98
MVT	2021	88.62
TransFER	ICCV 2021	90.91
APViT	IEEE Trans 2022	91.98
POSTER	ICCV 2022	92.05
QOT	IEEE Trans 2023	89.97
LCFC	Int. J. Intell. Syst. 2024	89.23
Baseline	-	91.88
TriCAFFNet	-	92.17

**Table 2:** Comparison results with other SOTA FER algorithms on AffectNet (7 cls).

Methods	Year	Accuracy
MVT	2021	64.57
TransFER	ICCV 2021	66.23
EAC	ECCV 2022	65.32
APViT	IEEE Trans 2022	66.91
POSTER	ICCV 2022	67.31
QOT	IEEE Trans 2023	67.37
Baseline	-	66.34
TriCAFFNet	-	67.40

**Table 3:** Comparison results with other SOTA FER algorithms on AffectNet (8 cls).

Methods	Year	Accuracy
PSR	2020	60.68
MVT	2021	61.40
TransFER	ICCV 2021	90.91
ARM	2021	61.33
POSTER	ICCV 2022	63.34
LCFC	Int. J. Intell. Syst. 2024	62.17
Baseline	-	63.14
TriCAFFNet	-	63.49

**Table 4:** Comparison of parameters, FLOPs, and accuracy on RAF-DB and AffectNet datasets with SOTA models.

Methods	Params	FLOPs	Acc (RAF-DB)	Acc (AffectNet 7 cls)
MVT	33.0 M	5.95 G	88.62	64.57
VTFF	51.8 M	6.08 G	88.14	61.85
TransFER	65.2 M	15.3 G	90.91	66.23
POSTER	71.8 M	15.7 G	92.05	67.31
APViT	65.2 M	12.67 G	91.98	66.91
Baseline	33.39 M	6.24 G	91.88	66.34
TriCAFFNet	32.8 M	6.7 G	92.17	67.40

**Table 5:** Comparison of class-wise accuracy with some SOTA models on RAF-DB.

Methods	Neutral	Happy	Sad	Surprise	Fear	Disgust	Anger	Mean Acc
MVT	89.12	95.61	87.45	87.54	60.81	63.75	78.40	80.38
VTFF	87.50	94.90	87.42	85.41	64.86	68.12	85.80	81.86
TransFER	90.15	95.95	88.70	89.06	68.92	79.37	88.89	85.86
APViT	90.06	97.30	88.70	93.31	72.97	73.75	86.42	86.36
Baseline	91.03	97.05	92.05	90.58	64.86	75.62	86.42	85.37
TriCAFFNet	92.94	96.79	91.42	89.97	70.27	74.38	88.27	86.29

**Table 6:** Comparison results of misclassification rate on RAF-DB (↓ indicates that TriCAFFNet has a lower misclassification rate than POSTER in this category, ↑ indicates that TriCAFFNet has a higher recognition rate than POSTER in this category).

Methods	Target	Prediction Percentage						
		Happy	Sad	Surprise	Fear	Disgust	Anger	Mean Acc
POSTER	Neutral	92.35	1.91	4.26	1.32	0.00	0.25	0.00
TriCAFFNet	Neutral	92.94 ↑	1.47 ↓	4.26	1.17 ↓	0.00	0.14 ↓	0.00
POSTER	Fear	4.05	2.70	9.46	12.16	67.57	2.70	1.35
TriCAFFNet	Fear	2.70 ↓	2.70	9.46	13.51	70.27 ↑	0.00 ↓	1.35
POSTER	Sad	5.23	1.26	91.21	0.63	0.00	1.46	0.21
TriCAFFNet	Sad	5.86	0.63 ↓	91.42 ↑	0.21 ↓	0.21	1.46	0.21

**Table 7:** Results of ablation experiments of key components of TriCAFFNet.

Methods	Acc (RAF-DB)	Acc (AffectNet)
TriCAFFNet	92.17	67.40
w/o landmark	91.40	67.10
w/o LBPHOG	91.74	67.30
w/o tri-cross-attention	91.39	67.14

## Data Availability

The original contributions presented in the study are included in the article, further inquiries can be directed to the corresponding author.
